# The impact of *Wolbachia* infection on the rate of vertical transmission of dengue virus in Brazilian *Aedes aegypti*

**DOI:** 10.1186/s13071-017-2236-z

**Published:** 2017-06-17

**Authors:** Etiene Casagrande Pacidônio, Eric Pearce Caragata, Debora Magalhães Alves, João Trindade Marques, Luciano Andrade Moreira

**Affiliations:** 1 Grupo Mosquitos Vetores: Endossimbiontes e Interação Patógeno Vetor, Centro de Pesquisas René Rachou – Fiocruz, Belo Horizonte, Minas Gerais Brazil; 2 Department of Biochemistry and Immunology, Instituto de Ciências Biológicas, Universidade Federal de Minas Gerais, Belo Horizonte, Minas Gerais Brazil; 3 Current address: School of Biological Sciences, Monash University, Clayton, Melbourne, VIC Australia

**Keywords:** *Wolbachia*, Dengue virus, *Aedes aegypti*, Vertical transmission, Arbovirus

## Abstract

**Background:**

*Wolbachia pipientis* is a common endosymbiotic bacterium of arthropods that strongly inhibits dengue virus (DENV) infection and transmission in the primary vector, the mosquito *Aedes aegypti*. For that reason, *Wolbachia*-infected *Ae. aegypti* are currently being released into the field as part of a novel strategy to reduce DENV transmission. However, there is evidence that DENV can be transmitted vertically from mother to progeny, and this may help the virus persist in nature in the absence of regular human transmission. The effect of *Wolbachia* infection on this process had not previously been examined.

**Results:**

We challenged *Ae. aegypti* with different Brazilian DENV isolates either by oral feeding or intrathoracic injection to ensure disseminated infection. We examined the effect of *Wolbachia* infection on the prevalence of DENV infection, and viral load in the ovaries. For orally infected mosquitoes, *Wolbachia* decreased the prevalence of infection by 71.29%, but there was no such effect when the virus was injected. Interestingly, regardless of the method of infection, *Wolbachia* infection strongly reduced DENV load in the ovaries. We then looked at the effect of *Wolbachia* on vertical transmission, where we observed only very low rates of vertical transmission. There was a trend towards lower rates in the presence of *Wolbachia*, with overall maximum likelihood estimate of infection rates of 5.04 per 1000 larvae for mosquitoes without *Wolbachia*, and 1.93 per 1000 larvae for *Wolbachia*-infected mosquitoes, after DENV injection. However, this effect was not statistically significant.

**Conclusions:**

Our data support the idea that vertical transmission of DENV is rare in nature, even in the absence of *Wolbachia*. Indeed, we observed that vertical transmission rates were low even when the midgut barrier was bypassed, which might help to explain why we only observed a trend towards lower vertical transmission rates in the presence of *Wolbachia.* Nevertheless, the low prevalence of disseminated DENV infection and lower DENV load in the ovaries supports the hypothesis that the presence of *Wolbachia* in *Ae. aegypti* would have an effect on the vertical transmission of DENV in the field.

**Electronic supplementary material:**

The online version of this article (doi:10.1186/s13071-017-2236-z) contains supplementary material, which is available to authorized users.

## Background

Novel mosquito control methods are required to reduce the heavy disease burden associated with dengue virus (DENV), with an estimated 390 million cases occurring annually [1]. One promising strategy involves using the bacterium *Wolbachia pipientis*, one of the most prevalent natural bacterial endosymbionts of insects [2]. Although the primary DENV vector, *Aedes aegypti*, is not naturally infected by *Wolbachia*, a stable infection with the *w*Mel *Wolbachia* strain have been generated *via* transinfection [3]. These *Wolbachia*-infected *Ae. aegypti* are highly resistant to DENV infection, exhibiting decreased prevalence of infection and DENV load, as well as decreased rates of disseminated infection [3, 4]. Critically, *w*Mel infection also greatly decreases viral prevalence in the salivary glands and more importantly the saliva, and therefore likely reduces vector competence for DENV [3–5].

This anti-DENV effect forms the basis of the *Wolbachia* transmission blocking approach, which is currently being used around the world to reduce the DENV burden (www.eliminatedengue.com). In this strategy, *w*Mel-infected mosquitoes are released into the field, where the bacterium can spread to high levels in wild populations due to cytoplasmic incompatibility, a type of reproductive incompatibility that favours the propagation of *Wolbachia*-infected mosquitoes and acts as a form of drive [6]. The widespread deployment of *w*Mel-infected mosquitoes in disease-endemic areas could potentially lead to a reduction in the transmission of DENV, or other viruses such as Zika and chikungunya [7–10].

Vertical transmission (VT) of DENV and other arboviruses from mother to progeny transovarially during egg development has been proposed as a potential explanation for the persistence of these viruses in nature during inter-epidemic periods [11–13]. Data suggest that this occurs only rarely, and the epidemiological importance is still unclear [12]. Given the impact of *Wolbachia* on viral dissemination, which likely affects viral access to the ovaries, we theorised that the anti-viral effects of the bacterium could also extend to VT. To that end, we sought to characterise the impact of *w*Mel infection on DENV VT, and DENV infection in the ovaries of Brazilian *Ae. aegypti*.

## Methods

### Mosquito lines and DENV isolates

Two *Ae. aegypti* mosquito lines were used in these experiments. The first, infected with the *w*Mel *Wolbachia* strain (+Wolb), was derived from the original *w*Mel-transinfected line [3] and backcrossed to a Brazilian genetic background, as previously described [14]. The second (-Wolb) was a *Wolbachia*-uninfected line, derived from +Wolb by tetracycline treatment, which occurred 2–3 years prior to the experiments described herein [14]. Microbial recolonization and outcrossing regimes for these lines have been previously described [14, 15].

Three DENV isolates were used in these experiments: DENV-1 BR-90 (6 × 10^5^ pfu/ml; isolated in Rio de Janeiro, RJ, Brazil, 1990, GenBank AF226685) [16], DENV-3 MG20 (1.9 × 10^6^ pfu/ml; MG20, isolated in Contagem, MG, Brazil, 2013, viral genome unsequenced), and DENV-4 Boa Vista 1981 (6 × 10^6^ pfu/ml; isolated in Boa Vista, RR, Brazil, 1981) [17]. Viruses were serially passaged in *Aedes albopictus* C6/36 cells, and infected supernatant harvested, titered *via* plaque forming assay, and then frozen, as previously described [15]. Virus aliquots were only thawed immediately prior to infection. All raw data from experiments are presented in Additional file 1.

### Oral infection

Four to seven day-old +Wolb and -Wolb females were starved overnight, and then orally infected by feeding DENV-4 mixed 1:1 with freshly drawn human blood. Blood-fed mosquitoes were maintained on 10% sucrose for 16 days to exceed the extrinsic incubation period (EIP) for DENV, allowing the virus to invade tissues such as the ovaries, thus increasing the chance for the viral invasion of developing eggs, and vertical transmission. A period longer than 14 dpi was selected as *w*Mel has been demonstrated to increase the duration of the EIP [18].

At this point, mosquitoes were offered a second, virus-free blood meal in order to induce egg development. Mosquitoes were allowed 3 days to lay eggs on damp filter paper in individual plastic Petri dishes and were then collected for quantification of DENV. The eggs laid by these mosquitoes were dried for 3 days and then hatched, and larvae reared to L_3_ and L_4_, whereupon they were washed twice with sterile water, dried on sterile filter paper, and then collected in pools containing an average of 7.79 larvae, with all larvae in each pool derived from a single female mosquito.

Total RNA from these samples was extracted using the TRIzol protocol (ThermoFisher Scientific, Waltham, MA, USA). DENV prevalence (proportion of mosquitoes infected) and viral load (number of DENV copies per 1 μg of total RNA) of infection were quantified for adults and larvae using *Taq*Man-based RT-qPCR using a Viia 7 Real-Time PCR System (ThermoFisher Scientific, Waltham, MA, USA) and the following primers and probe: DENV_F: (5′-AAG GAC TAG AGG TTA GAG GAG ACC C-3′), DENV_R: (5′-CGT TCT GTG CCT GGA ATG ATG-3′), Probe: (5′-HEX- AAC AGC ATA TTG ACG CTG GGA GAG ACC AGA -BHQ1–3′). RNA samples were quantified in duplicate using the *Taq*Man Fast Virus 1-Step Master Mix (ThermoFisher Scientific, Waltham, MA, USA), using a run profile of 50 °C for 6 min for reverse transcription, followed by 95 °C for 20 min to denature the enzyme, and then 45 cycles of 95 °C for 3 s, 60 °C for 30 s, and 72 °C for 1 s. For quantification purposes, the sequence of the DENV amplicon was cloned, amplified and then serially diluted to generate a standard curve, as previously described [19]. DENV positive +Wolb pools were screened for the presence of *Wolbachia* using the *wd0513* gene [8], as described above.

In a separate experiment following the protocol described above, the ovaries of DENV-challenged mosquitoes were dissected in sterile 1× PBS 3 days after the second blood meal, total RNA was extracted, and RT-PCR performed as above.

### Intrathoracic injection

Four to seven day-old -Wolb and +Wolb female mosquitoes were injected intrathoracically with 69 nl of DENV-1, DENV-3, or DENV-4 using a Nanoject II injector (Drummond Scientific, Broomall, PA, USA). Virus stocks were not diluted from the concentrations described above. Seven days later, mosquitoes were fed a virus-free blood meal in order to stimulate egg production, and then larval collection was performed as above, with pools containing an average of 4.19 larvae. The decreased pool size was reflective of lower adult fecundity rates post-injection. Five experiments were performed: 3 using DENV-4, and 1 each for DENV-1 and DENV-3. Ovaries were dissected from mosquitoes in one of the DENV-4 experiments, and the DENV-1 experiment, and processed as above.

### Data analysis

In each experiment, the maximum likelihood estimate (MLE) of infection rate per 1000 larvae was calculated independently for -Wolb and +Wolb mosquitoes using the formula:

$$ \mathrm{MLE}=1-{\left(1- Y/ X\right)}^{1/ m} $$

where *Y* is the number of positive pools, *X* is the total number of pools, and *m* is the average pool size in the experiment [20].

Minimum infection rates (MIR) were not calculated due to non-uniformity in pool sizes. MLEs were also compiled across all injection data, and compared between -Wolb and +Wolb mosquitoes using Fisher’s exact test.

DENV prevalence data (proportion of mosquitoes infected) were compared between -Wolb and +Wolb mosquitoes using Fisher’s exact test, while DENV load data (DENV titre) were not normally distributed, and were compared using Mann-Whitney U-tests. All statistical analyses were performed using Prism 6.0 g (Graphpad).

## Results and discussion

### Ovary infection

**Fig. 1:**
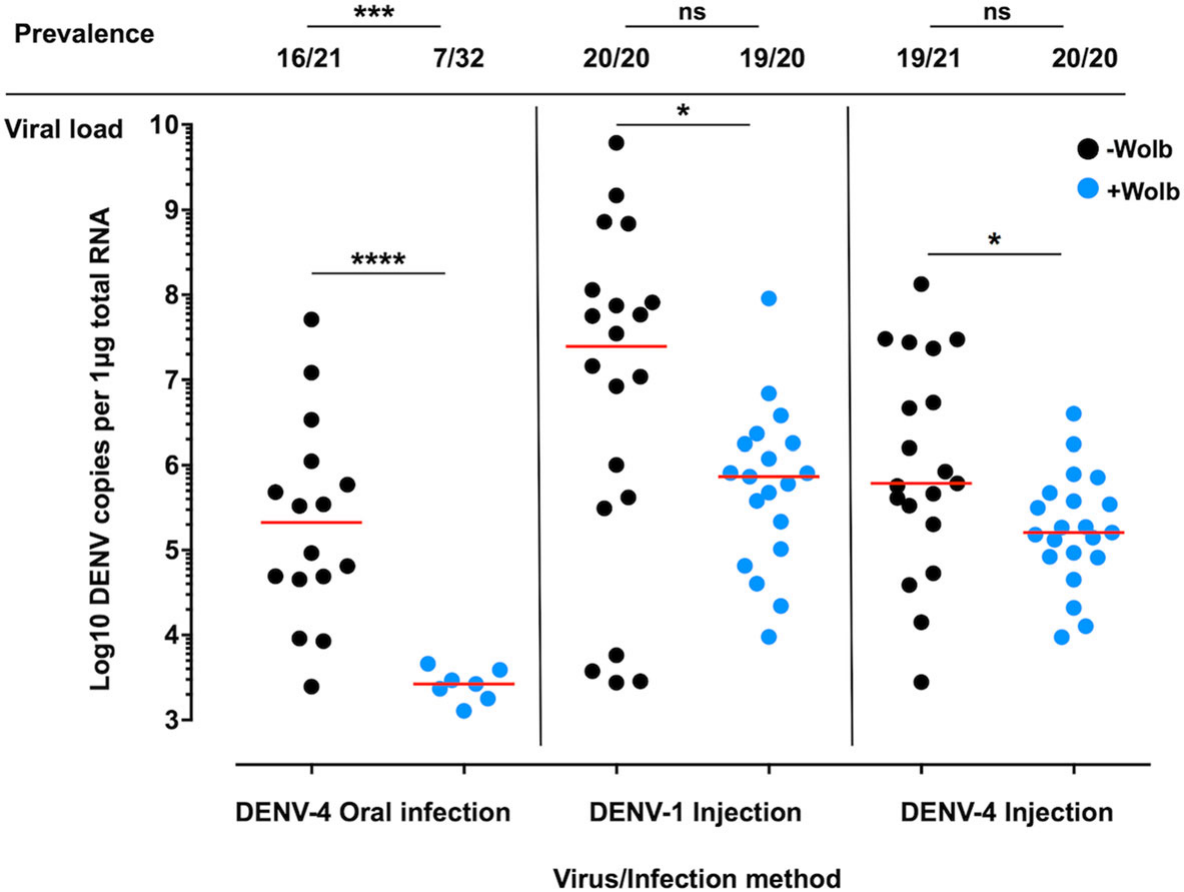
The effect of *w*Mel on DENV infection in *Aedes aegypti* ovaries. DENV prevalence and viral load in the ovaries of mosquitoes with (+Wolb, *blue circles*) and without (-Wolb, *black circles*) *w*Mel *Wolbachia* infection. Mosquitoes were challenged with DENV-4 orally or *via* intrathoracic injection, or with DENV-1 *via* injection. Mosquitoes were fed on a virus-free blood meal either 16 days after DENV oral infection or 7 days post-DENV injection, with these times corresponding to the blood meal used to induce egg production in our vertical transmission experiments. At 72 h post-feeding, ovaries were dissected in sterile 1× PBS, total RNA was extracted, and genomic DENV levels quantified *via* RT-qPCR. Prevalence data were compared by Fisher’s exact test (***P < 0.001, ns: *P* > 0.05). Viral load data were compared pairwise by Mann-Whitney U-test (****P < 0.0001, *P < 0.05). Horizontal *red lines* represent treatment medians

DENV infection in the ovaries is a likely stepping-stone to VT [12]. In ovaries dissected from -Wolb and +Wolb mosquitoes that were orally challenged with DENV-4, we observed 71.29% decrease in prevalence of infection associated with *w*Mel (Fig. 1, Fisher’s exact test: *P* = 0.0002, OR = 0.0875, CI = 0.0237–0.327), which could indicate that the anti-DENV effect of *Wolbachia* can operate on the tissue level, or that the general inhibitory effect of *Wolbachia* limits the amount of virus that reaches the ovaries [21]. Critically, this effect disappeared after challenge with DENV-1 or DENV-4 *via* injection (Fisher’s exact test: DENV-1: *P* = 1, OR = 0.3171, CI = 0.0122–8.267; DENV-4: *P* = 0.4878, OR = 5.513, CI = 0.2488–122.2), highlighting the importance of the midgut to the anti-DENV effects of *w*Mel.

However, regardless of the method of viral challenge, we observed that significantly decreased DENV load in the ovaries was associated with the presence of *w*Mel (Mann-Whitney U-tests: DENV-4 oral infection: *U* = 4, *n*_1_ = 16, *n*_2_ = 7, *P* < 0.0001; DENV-1 injection: *U* = 116, *n*_1_ = 20, *n*_2_ = 19, *P* = 0.0379; DENV-4 injection: *U* = 112, *n*_1_ = 19, *n*_2_ = 21, *P* = 0.0173). This equated to a reduction in median DENV load of 98.75% in the oral feeding experiment, and a 97.04% and 73.69% decrease in the levels of DENV-1 and DENV-4 injection experiments, respectively. This decrease in viral load was not particularly surprising given the correlation between bacterial density and the presence of the antiviral effect [22, 23], and the fact that the ovaries typically have the highest *Wolbachia* density of any tissue, in order to facilitate high rates of maternal transmission [3, 24].

### Vertical transmission

We observed low parental infection rates in the DENV-4 oral infection experiment, with a significant reduction associated with *w*Mel infection (-Wolb: 11/60, +Wolb: 2/81; Fisher’s exact test, *P* = 0.002, OR = 0.1128, CI = 0.0240–0.5306). A total of 713 -Wolb larvae and 556 +Wolb larvae were screened in 85 and 78 pools, respectively, with only a single positive pool identified in the -Wolb treatment. This equated to MLE infection rates of 1.41 and 0 for -Wolb and +Wolb mosquitoes, respectively (Table 1). These VT rates were similar to those seen in other studies of the VT of DENV and other arboviruses, with the implication being that overall VT rates are low after DENV oral infection, and therefore low in nature [25].

**Table 1:** DENV vertical transmission rates and maximum likelihood of infection estimates

Experiment	Virus	Wolb	PPI	Larvae	ALPP	DENV-positive pools/total pools	MLE IR
Oral feeding							
1	DENV-4	−	0.18	713	8.39	1/85	1.41
		+	0.02	556	7.13	0/78	0
Injection							
2	DENV-1	−	1.00	277	4.07	1/68	3.63
		+	0.96	388	4.51	0/86	0
3	DENV-3	−	1.00	168	4.42	0/38	0
		+	0.97	371	4.52	0/82	0
4	DENV-4	−	1.00	274	4.35	3/63	11.16
		+	1.00	52	3.47	1/15	19.71
5	DENV-4	−	1.00	113	4.71	0/24	0
		+	0.96	361	4.40	2/82	5.59
6	DENV-4	−	1.00	168	2.67	1/63	5.98
		+	1.00	385	4.33	0/89	0
All experiments	All viruses	−	1.00	1000	3.91	5/256	5.04
		+	0.98	1557	4.40	3/354	1.93

*Abbreviations*: *DENV VT* vertical transmission of dengue virus rate; *Wolb Wolbachia* infection status; +, infected; −, uninfected; *PPI* proportion of parents infected; Larvae, number of larvae; *ALPP*, average larvae per pool; *MLE IR* maximum likelihood estimate of infection rate per 1000 larvae; All, data averaged across the 5 injection experiments

Rather than continuing with oral infections, we decided to bypass the midgut barrier and challenge mosquitoes by intrathoracic injection of DENV-1, -3 or -4 to ensure disseminated infection, thereby increasing the chance of VT. Across the five experiments that we performed, the average prevalence was 1.00 for -Wolb mosquitoes, and 0.98 for +Wolb mosquitoes (Fisher’s exact test, *P* = 0.2605, OR = 0.1829, CI = 0.0093–3.583), indicating that the midgut barrier was a key component in the anti-DENV effects of *w*Mel in Brazilian *Ae. aegypti*. In comparison, similar injection experiments with the *w*MelPop strain still demonstrated an anti-viral effect [19].

In spite of this increase in parental DENV prevalence, VT rates were low regardless of the DENV isolate used, or mosquito *Wolbachia* infection status (Table 1). We found five positive -Wolb pools out of a total of 256 (DENV-1: 1 pool; DENV-4: 4 pools), and three positive +Wolb pools out of 354 (all DENV-4), each of which was associated with a different adult mosquito. Accordingly, MLEs varied greatly between experiments. In two experiments the MLE was higher for +Wolb mosquitoes. This was likely due to the low number of samples collected, with only 15 +Wolb pools collected in the first of these experiments, and 24 -Wolb pools collected without any detectable DENV in the second.

Amalgamated MLEs were calculated for -Wolb and +Wolb across these five experiments in order to gain greater insight into VT rates across a larger data set. As expected, these data indicated that VT rates associated with DENV injection were slightly higher than those seen for DENV oral feeding. The -Wolb amalgamated MLE was 2.60 times higher than the MLE for +Wolb mosquitoes. However, this trend towards lower VT rates associated with *w*Mel infection was not statistically significant, potentially due to the very low frequency of observance (Fisher’s exact test: *P* = 0.29, OR = 0.4291, CI = 0.1016–1.812). We then screened each of the three +Wolb pools that were positive for DENV for the presence of the *Wolbachia wd0513* gene. Two pools were positive for *Wolbachia*, however we could not detect *Wolbachia* in one of the pools from DENV-4 experiment two. The mother of this pool was found to be positive for *Wolbachia*, which suggested that the negative result was either due to incomplete maternal transmission, or technical error. This sample was a pool of five larvae, and given that the maternal transmission rate of *w*Mel is around 99% [14], it is very unlikely that all five larvae were uninfected by *Wolbachia*. As these pools were screened for the presence of *Wolbachia* 2.5 years after the initial RNA extraction and DENV quantification was performed, the age of the material may have contributed to the failure to detect *Wolbachia* in that sample.

### Implications for VT in the field

Previous data have indicated that *w*Mel infection leads to an estimated 42% reduction in the prevalence of abdominal DENV infection 14 days after oral infection [4]. The implication of this is that a *Wolbachia* infection in a mosquito population would greatly reduce the number of individual mosquitoes that could potentially transmit DENV to their progeny. What our data suggest is that for those *w*Mel-infected mosquitoes that do become infected, the presence of the bacterium in the ovaries may serve as a further impediment to viral growth in this organ. Under these circumstances, it is quite likely that the widespread presence of *w*Mel-infected *Ae. aegypti* in nature would decrease the rate of transovarial transmission of DENV. It is also possible that VT of DENV may occur without viral infection of the ovaries. Our data shows that the presence of *Wolbachia* affects ovary infection to a greater extent than its impact on VT, which might suggest that these processes are not connected. If this were true, a potential transovarial-independent mechanism of VT could show less sensitivity to the anti-DENV effect of *Wolbachia*.

## Conclusions

We observed that overall VT rates were extremely low, even when the midgut barrier was bypassed to increase viral dissemination rates and the probability of ovary infection. In that sense, our results support the conclusion that VT in the field is quite rare and likely of little epidemiological consequence. However, a more epidemiologically relevant method of determining this would be to examine whether progeny that become infected by VT are capable of transmitting DENV as adults. In our experiments, we observed a trend towards reduced VT rates in *Ae. aegypti* due to the presence of *w*Mel although this was not statistically significant, likely due to the low overall frequency of VT. Interestingly, we did observe that *w*Mel infection greatly decreased the prevalence of infection and DENV load in mosquito ovaries, which would likely have a significant impact on transovarial VT rates in large mosquito populations in nature. This effect could represent a further benefit associated with the use of *Wolbachia*-infected *Ae. aegypti* for DENV control.

## Additional file

Additional file 1: Summary descriptions of all experiments, including DENV infection rates, DENV copies for mosquito adults and larvae, and fecundity counts (XLSX 69 kb)

## Abbreviations

+Wolb: *w*Mel-infected *Aedes aegypti*
DENV: Dengue virus
DENV-1/DENV-3/DENV-4: Dengue virus serotypes 1, 3 or 4
dpi: Days post-infection
EIP: Extrinsic incubation period
MIR: Minimum infection rate
MLE: Maximum likelihood estimate of infection rate
pfu: Plaque-forming units
RNA: Ribonucleic acid
RT-qPCR: Quantitative reverse transcription polymerase chain reaction
VT: Vertical transmission
-Wolb: *Aedes aegypti* uninfected by *Wolbachia*
